# Supplementary material for: Unbiased measurements of reconstruction fidelity of sparsely sampled magnetic resonance spectra
Source: Nat Commun. 2016 Jul 27;7:12281. doi: 10.1038/ncomms12281 (PMC4974455; doi:10.1038/ncomms12281)
Supplement: Supplementary Information — Supplementary Figures 1-6. [file ncomms12281-s1.pdf]

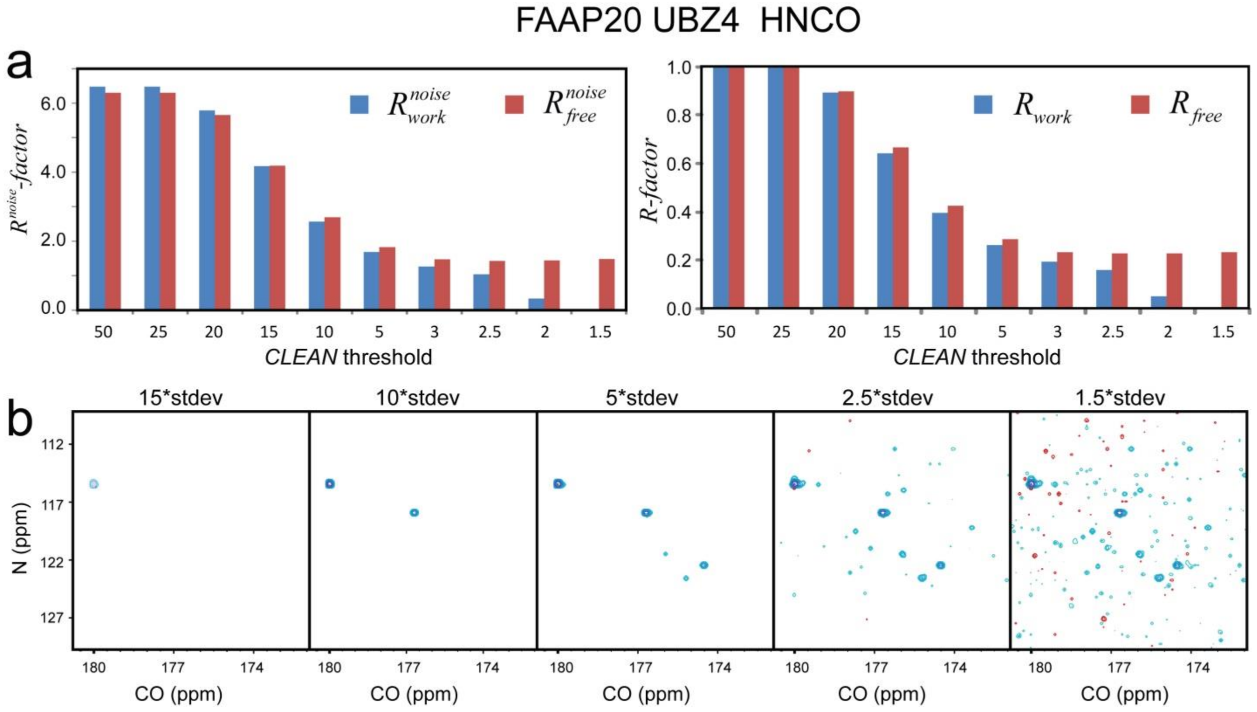

Supplementary Figure 1 | Quality measurements of an N-CO plane of the reconstructed 3-D HNCO spectrum of FAAP20 UBZ4 by CLEAN. (**a**) Progression of the quality factors R^{noise} (left) and R (right) during the CLEAN reconstruction of an N-CO plane at HN of 8.27 ppm. (**b**) Modeled components by CLEAN at different stopping thresholds.

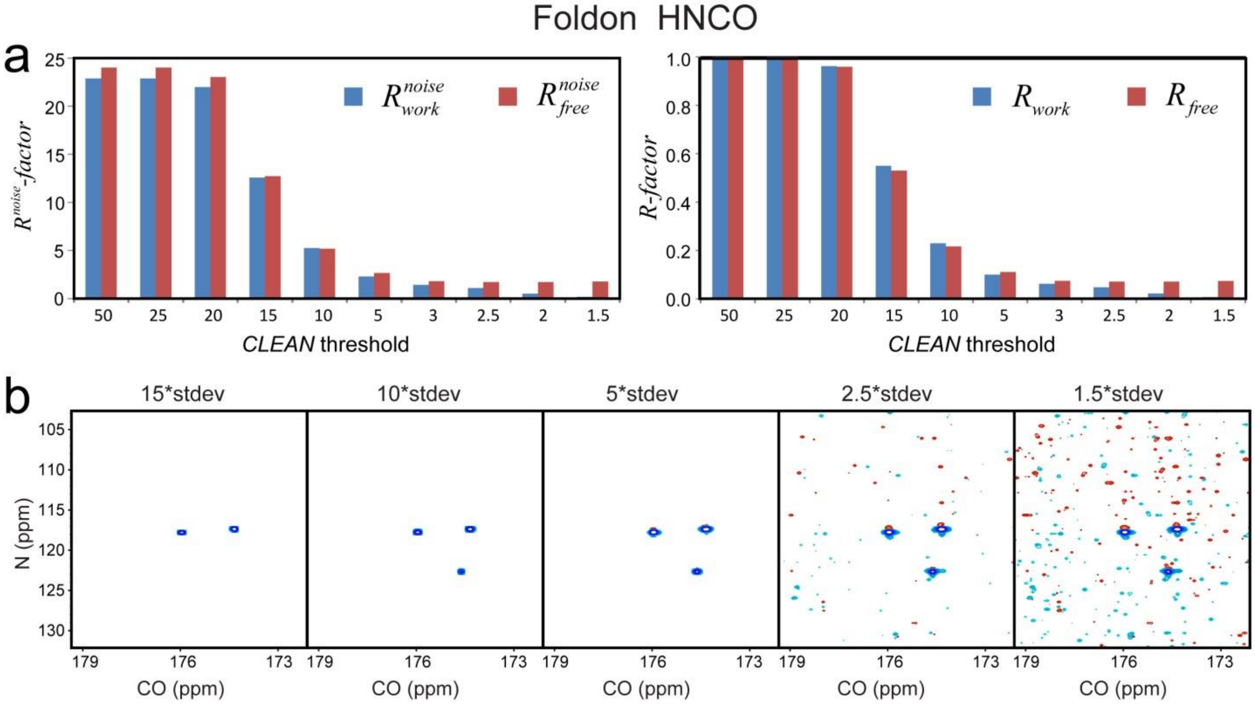

Supplementary Figure 2 | Quality measurements of an N-CO plane of the reconstructed 3-D HNCO spectrum of foldon by CLEAN. (**a**) Progression of the quality factors R^{noise} (left) and R (right) during the CLEAN reconstruction of an N-CO plane at HN of 8.32 ppm. (**b**) Modeled components by CLEAN at different stopping thresholds.

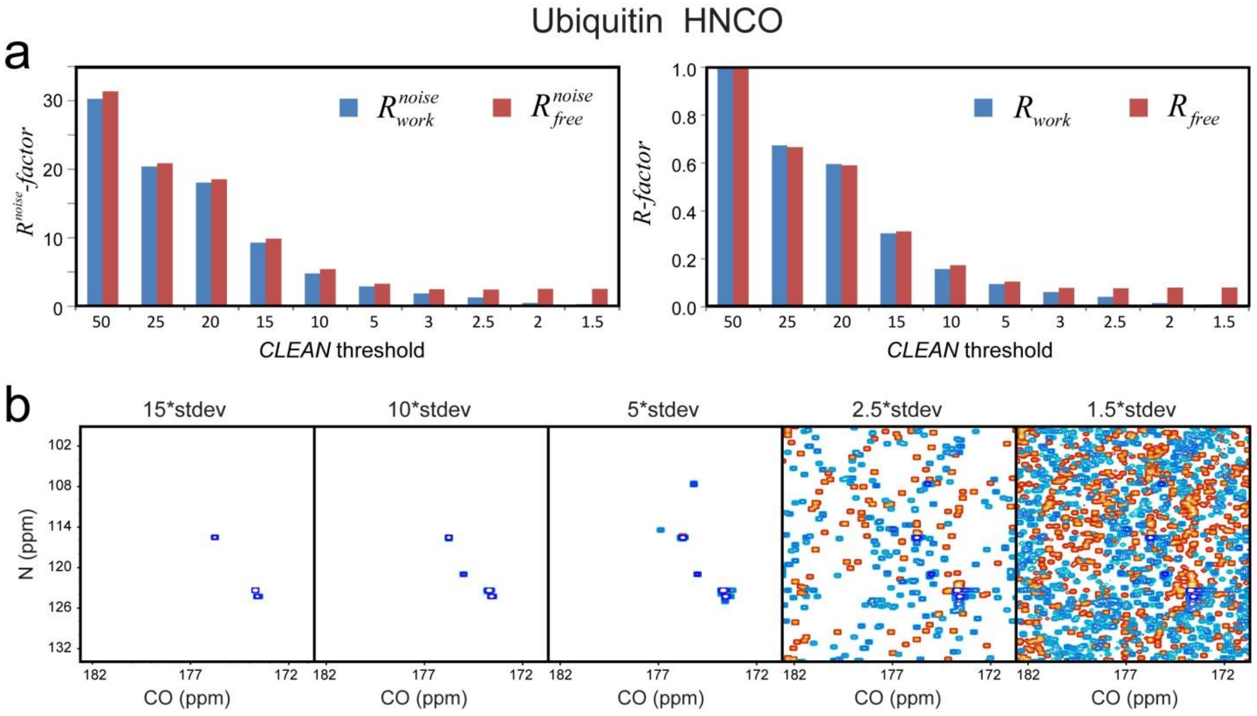

Supplementary Figure 3 | Quality measurements of an N-CO plane of the reconstructed 3-D HNCO spectrum of ubiquitin by CLEAN. (**a**) Progression of the quality factors R^{noise} (left) and R (right) during the CLEAN reconstruction of an N-CO plane at HN of 8.82 ppm. (**b**) Modeled components by CLEAN at different stopping thresholds.

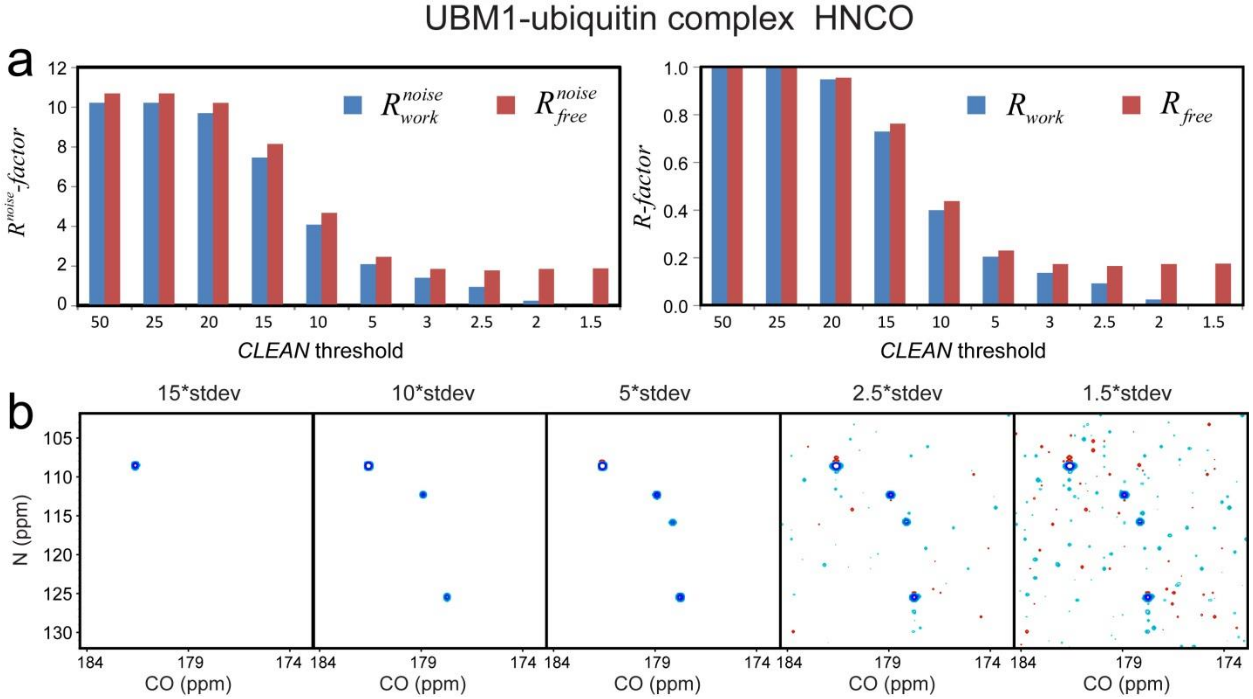

Supplementary Figure 4 | Quality measurements of an N-CO plane of the reconstructed 3-D HNCO spectrum of the UBM1-ubiquitin complex by CLEAN. (**a**) Progression of the quality factors R^{noise} (left) and R (right) during the CLEAN reconstruction of an N-CO plane at HN of 8.77 ppm. (**b**) Modeled components by CLEAN at different stopping thresholds.

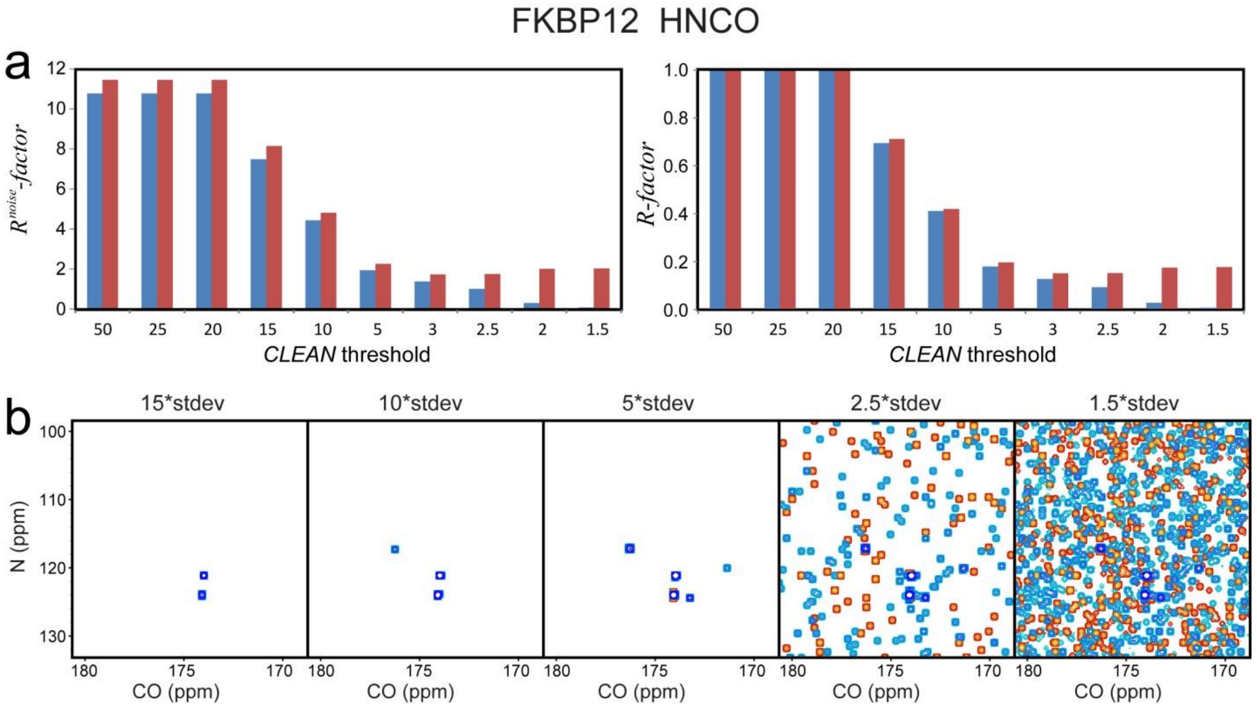

Supplementary Figure 5 | Quality measurements of an N-CO plane of the reconstructed 3-D HNCO spectrum of FKBP12 by CLEAN. (**a**) Progression of the quality factors R^{noise} (left) and R (right) during the CLEAN reconstruction of an N-CO plane at HN of 8.72 ppm. (**b**) Modeled components by CLEAN at different stopping thresholds.

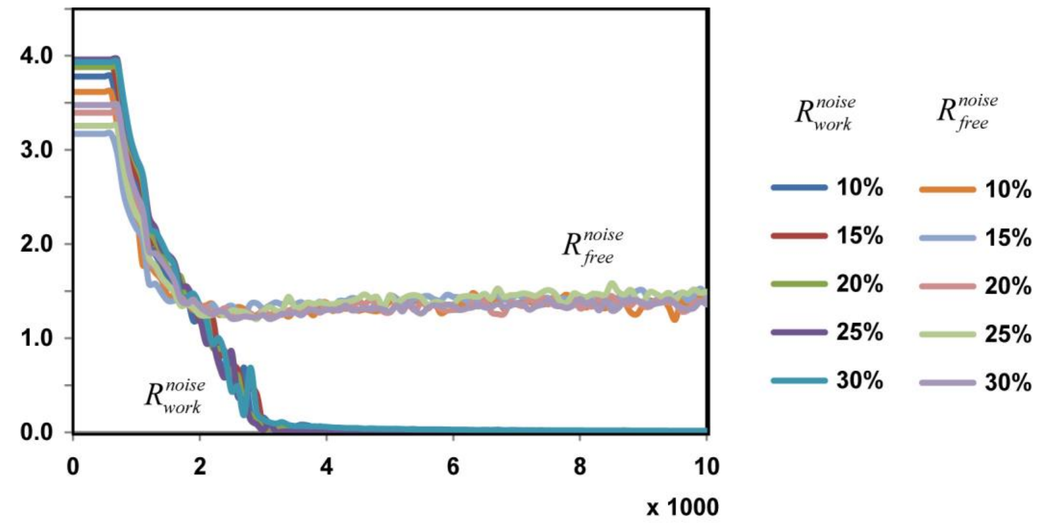

Supplementary Figure 6 | Stability of R^{noise} factors using 10-30% of measurements for cross-validation.
